# Comparing Different Approaches of (Not) Accounting for Rapid Guessing in Plausible Values Estimation

**DOI:** 10.1177/00131644251395590

**Published:** 2026-01-13

**Authors:** Jana Welling, Eva Zink, Timo Gnambs

**Affiliations:** 1 Leibniz Institute for Educational Trajectories, Bamberg, Germany

**Keywords:** rapid guessing, plausible values, large-scale assessments, test-taking engagement, engagement gap, ability comparisons

## Abstract

Educational large-scale assessments provide information on ability differences between groups, informing policies and shaping educational decisions. However, some of these differences might partly reflect variations in test-taking motivation rather than in actual abilities. Existing approaches for mitigating the distorting effects of rapid guessing focus mainly on point estimates of abilities, although research questions often refer to latent variables. The present study seeks to (a) determine the bias introduced by rapid guessing in group comparisons based on plausible value estimates and (b) introduce and evaluate different approaches of handling rapid guessing in the estimation of plausible values. In a simulation study, four models were compared: (1) a baseline model did not account for rapid guessing, (2) a person-level model incorporated rapid guessing as a respondent characteristic in the background model, (3) a response-level model filtered responses with item response times lower than a predetermined threshold, and (4) a combined model merged the person- and response-level approaches. Results show that the response-level and combined model performed best while accounting for rapid guessing on the person level did not suffice. An empirical example using data from a German large-scale assessment (*N* = 478) demonstrates the applicability of all approaches in practice. Recommendations for future research are given to improve ability estimation.

Educational large-scale assessments (LSAs) are designed to evaluate domain-specific competencies and their differences across relevant groups such as countries, educational institutions, or gender. Prominent LSAs like the *Programme for International Student Assessment* (PISA), *Programme for the International Assessment of Adult Competencies* (PIAAC), and *Trends in International Mathematics and Science Study* (TIMSS) typically use plausible values (PVs) to account for measurement error and correct for sampling design in the estimation of abilities to provide unbiased estimates of population parameters (e.g., Mislevy, 1991; Wu, 2005). Thus, they enable valid inferences about populations and subgroups, rather than providing precise estimates of individual abilities, making them a cornerstone of LSAs.

LSAs are typically low stakes because test results do not have direct consequences for the participants. Consequently, some test-takers may lack sufficient motivation or exert minimal effort when completing these tests, leading to rapid guessing (RG)—a response behavior characterized by superficially quick item responses that do not reflect actual knowledge or abilities (Wise & DeMars, 2005). Because responses influenced by RG do not reflect the actual proficiency of the test-takers, they pose a threat to the validity of ability estimates (Wise, 2017). Simulation studies have shown that RG introduces substantial biases in point estimates of proficiency and has a nonnegligible impact across diverse testing contexts (e.g., DeMars et al., 2013; Osborne & Blanchard, 2011; Wise & DeMars, 2010). However, while PVs are commonly used in educational LSAs, the extent to which RG affects PV estimates has not been systematically explored. Given that PVs reflect uncertainty at the population level rather than for individual respondents, RG may primarily affect analyses of aggregated data, for example, on differences between groups.

This study therefore aimed to investigate the effect of RG on PV estimates and to evaluate methods for addressing it in the context of ability comparisons between groups (hereinafter referred to as *group comparisons*). Specifically, we seek to (a) determine the bias introduced by RG in group comparisons based on PV estimates, and (b) introduce and evaluate different approaches of handling RG in the estimation of PVs. To address these research aims, we first present different methods of accounting for RG in PV estimation. In a simulation study and an illustrative application using data from university students participating in a German LSA, we determine to what extent RG biases group comparisons based on PVs and evaluate the effectiveness of the different approaches in mitigating these biases.

## Rapid Guessing in Large-Scale Assessments

### Defining Rapid Guessing

Schnipke and Scrams (1997) classify test-taking behavior into two distinct categories: solution behavior (SB) and RG behavior. SB involves carefully analyzing a question while employing one’s abilities and effort after thoroughly reading the item. In contrast, RG behavior is characterized by quickly skimming the question and selecting an answer without meaningful engagement. As a result, responses produced with RG do not accurately reflect test-taker’s true abilities in the same way as those given under SB (Wise, 2017).

However, many existing IRT models are designed for SB by assuming that the probability of a correct response follows a monotonically increasing function depending on the respondent’s proficiency, meaning that higher proficiency levels correspond to higher probabilities of correct responses (C. Wang & Xu, 2015). For example, under the one-parametric logistic test model (Rasch, 1960), the probability of a correct response is given as

(1) $$P(Y_{\mathrm{ij}}=1|\theta_{i},\xi_{j})=\frac{\exp(\theta_{i}-\xi_{j})}{1+\exp(\theta_{i}-\xi_{j})}$$

with the binary response $Y_{\mathrm{ij}}$ given as a function of the ability parameter $\theta_{i}$ of person i and the difficulty parameter $\xi_{j}$ of item j . In contrast, under RG, the assumptions of the Rasch model do not hold as test-takers respond without considering the response options, relying purely on guessing. The observed responses are assumed to be independent of the test-taker’s true ability and thus provide no meaningful information about it (Schnipke & Scrams, 1997; C. Wang & Xu, 2015).

Reported RG rates in LSAs vary widely, ranging from under 1% (Kroehne et al., 2020) to nearly 20% (Ulitzsch et al., 2020), depending on factors such as test domain, testing conditions, and the identification method used (e.g., Goldhammer et al., 2016; Kroehne et al., 2020; Michaelides & Ivanova, 2022; Rios et al., 2017). Because RG occurs at the response level (Ulitzsch et al., 2020), it is influenced by both test-taker characteristics (e.g., ability; Rios & Soland, 2022) and item characteristics (e.g., placement, difficulty, and formatting; Lee & Jia, 2014). Typically, test-takers with higher ability levels exhibit lower rates of RG, and more difficult items tend to lead to higher RG rates (e.g., Goldhammer, Martens, & Lüdtke, 2017; Rios et al., 2022; Soland, 2018), highlighting the complex relationship between test-takers’ ability, RG, and item characteristics.

### Biases Introduced by Rapid Guessing

Failing to account for RG biases both item and person parameter estimates in models that assume SB (Jin et al., 2022; Rios et al., 2017; Rios & Soland, 2021a). The extent of this bias increases with higher RG rates (Rios & Soland, 2021a; Wise et al., 2021) and a greater difference between the probability of a correct response when engaging in SB and the probability of a correct guess when engaging in RG (which is mostly notable in easy tests, where the probability of a correct response for SB is generally high; Rios et al., 2017).

In addition, RG can distort ability comparisons between groups (Soland, 2018). For example, Anaya and Zamarro (2023) found that boys exhibit higher RG rates than girls in PISA assessments, independent of their ability level. Accounting for these differences substantially altered estimates of the gender achievement gap, shifting mathematics and science scores by up to 36% and 40% of a standard deviation, respectively, in favor of boys. These findings underscore the substantial threat RG poses to the validity of scores in general (e.g., DeMars, 2007; Setzer et al., 2013; Wise et al., 2009) and to group comparisons in particular (Anaya & Zamarro, 2023; Soland, 2018; Wise, 2017). They highlight the importance of robust methods to identify and adjust for RG when interpreting data from LSAs to ensure accurate interpretation of results.

### Measuring Rapid Guessing

RG can be measured at multiple analytical levels. At the examinee level, it is measured across all responses for each test-taker to assess individual differences in RG behavior (Wise & Kong, 2005). At the item level, RG patterns are aggregated across all test-takers for a given item because some items may provoke higher rates of RG than others (Schnipke & Scrams, 1997). Finally, at the response level, each interaction between a test-taker and an item is analyzed to identify specific responses from a test-taker as a rapid guess.

Although RG behavior cannot be directly observed, three primary proxies have been proposed for its measurement: (a) self-reported effort, (b) aberrant response patterns, and (c) response times (Rios & Deng, 2021). Of these, response time-based methods have gained particular popularity in recent years (e.g., Goldhammer, Naumann, et al., 2017; Silm et al., 2020; Wise & Kuhfeld, 2021) due to their effectiveness in classifying responses below a specified threshold as RG. By leveraging process data automatically collected in computerized assessments, this approach minimizes concerns about observer effects (Rios & Deng, 2021). Furthermore, it enables the evaluation of RG on the response level, facilitating detailed analyses of shifts in test-taking behavior across items (Wise & Kingsbury, 2016).

Methods of detecting and accounting for RG using response times can be broadly categorized into two groups: model-based and threshold-based approaches. Model-based approaches distinguish between SB and RG using mixture modeling techniques (Nagy & Ulitzsch, 2022; C. Wang & Xu, 2015). In these mixture models, the probability of a correct response depends on the response behavior. For responses identified as SB, the probability of a correct response is modeled using traditional IRT models, for example, the Rasch model as depicted in Equation 1. For responses identified as RG, however, the probability is set to chance level, assuming that these responses do not provide meaningful information about a test-takers’ ability (Schnipke & Scrams, 1997; C. Wang & Xu, 2015).

Research shows that model-based approaches effectively reduce RG bias by identifying and accounting for responses classified as RG (Rios et al., 2022). However, their practical application is limited by high computational demands, large sample size requirements, challenges with model convergence, and assumptions about response time distributions (Molenaar et al., 2018). Therefore, in practice, threshold-based approaches are most commonly used (Rios & Deng, 2021; Silm et al., 2020). These assume distinct response time distributions for SB and RG. By comparing an individual’s item response time $t_{\mathrm{ij}}$ against a predefined threshold $\rho_{j}$ of item *j*, the engagement $\phi_{\mathrm{ij}}$ for each item-person encounter is determined (Goldhammer, Martens, & Lüdtke, 2017). If the response time is shorter than the threshold, the response is flagged as RG, meaning

(2) $$\phi_{\mathrm{ij}}\in \left\{ \begin{array}{c} 0,\;\mathrm{if}\;t_{\mathrm{ij}}<\rho_{j}\\ \;1,\;\mathrm{if}\;t_{\mathrm{ij}}\geq \rho_{j.} \end{array}\right.$$

Therefore, the engagement $\phi_{\mathrm{ij}}$ takes the value 0 if the response is flagged as a rapid guess and 1 otherwise. However, this requires an accurate threshold determination, as inaccurate thresholds may also bias item and parameter estimates (Rios, 2022).

### Selection of a Threshold

Threshold-based approaches of accounting for RG require the selection of a response time threshold to classify responses as either RG or SB based on the assumption that the two behaviors correspond to distinct, nonoverlapping response time distributions (Rios & Deng, 2023). Thresholds can be determined through heuristic rules, examination of response time distributions, or the combination of response time with accuracy information (Rios & Deng, 2021). In the latter case, unusually short responses that are also incorrect are taken as strong indicators of RG, whereas longer response times accompanied by higher accuracy are more consistent with solution behavior (Rios & Deng, 2021). The following paragraphs introduce two well-established and easily applicable threshold techniques (Buchholz et al., 2022; Goldhammer et al., 2016; Kroehne et al., 2020; Michaelides & Ivanova, 2022): the common-*k* and the visual-inspection method.

The common-*k* or fixed threshold method applies a uniform response time threshold across all items (Wise et al., 2004) and is widely used due to its simplicity, including in LSAs like PIAAC (Goldhammer et al., 2016). Its main advantage is that it does not require item-specific data, making it suitable for large item pools with minimal effort. However, a key limitation is its failure to account for item variability. Applying a uniform threshold to items with, for example, different reading demands may overlook meaningful differences in response behavior. Although some research suggested that response time distributions remain nearly uniform under RG due to disengagement (C. Wang & Xu, 2015), the response time distributions under SB can vary between items and populations. This method may thus fail to account for heterogeneity in items (e.g., item difficulties) and population characteristics (e.g., person abilities), potentially undermining its validity (Wise, 2017).

The visual-inspection method proposed by Schnipke (1995) identifies response time thresholds based on the bimodal distribution of response times, where the first mode represents RG and the second reflects SB. The threshold is set at the lowest intersection point between these distributions. While this method is effective, it is also time-consuming and may lead to different interpretations among observers (Rios & Deng, 2021). The assumption that response times follow a bimodal distribution for every item does not always hold in practice, particularly for items that can be solved quickly even under SB (Wise, 2017). In addition, this method has the tendency to set excessively high thresholds, which may lead to misclassification of engaged responses as RG (e.g., Wise & Kuhfeld, 2020, 2021).

## Plausible Value Technique

PVs account for measurement error in the estimation of competencies while also correcting for sampling design to allow for unbiased population estimates (e.g., Mislevy, 1991; Wu, 2005). They were developed in the context of LSAs to enable valid inferences about populations and subgroups rather than to allow a precise measurement of an individual’s ability (e.g., von Davier et al., 2009; Wu, 2005). PVs are generated by drawing multiple estimates of an individual’s latent ability, thereby reflecting the range of abilities that are plausible for that person given their item responses (Wu, 2005). The PVs approach applies the concept of multiple imputation (Rubin, 1987; see also Jewsbury et al., 2024), wherein multiple values for each individual i are drawn from the posterior predictive distribution of their latent ability, $\theta_{i}$ (Mislevy, 1991). The posterior predictive distribution can be described as

(3) $$P\left(\theta_{i}|Y_{i},X_{i};\xi ,\psi \right)\propto P\left(Y_{i}|\theta_{i};\xi \right)\cdot P\left(\theta_{i}|X_{i};\psi \right),$$

with $Y_{i\;}$ representing the participant’s item responses, ξ the parameters of the measurement model, and $X_{i}$ the background variables. The posterior predictive distribution integrates two key components: the measurement model $P\left(Y_{i}|\theta_{i};\xi \right)$ , which establishes the relationship between the latent ability $\theta_{i}$ and the item responses $Y_{i\;}$ , and the background model $P(\theta_{i}|X_{i};\psi )$ , which describes how background variables $X_{i}$ relate to the ability $\theta_{i}$ (Mislevy, 1991; Mislevy et al., 1992). The measurement model is typically defined using an IRT model, which specifies the probability of a response pattern $Y_{i}\in \left\{y_{i1},\;\ldots ,\;y_{\mathrm{iJ}}\right\}$ for a person i∈{1,…,I} to a set of items j∈{1,…,J} as follows,

(4) $$P(Y_{i}|\theta_{i};\xi )=\overset{J}{\underset{j=1}{\Pi }}P(y_{\mathrm{ij}}|\theta_{i};\xi_{j}).$$

A commonly used IRT model is the Rasch model (Equation 1). The background model is typically parameterized as a linear regression as shown in Equation (4), where $\psi_{0}$ denotes the intercept, and $\psi_{X}$ represents a vector of regression coefficients for the predictors included in the background model

(5) $$P\left(\theta_{i}|X_{i};\psi \right)\propto N\left(\psi_{0}+\psi_{X}X_{i};\sigma_{\theta_{i}|X_{i}}^{2}\right)$$

An important challenge in PV estimation is ensuring that all relevant variables $X_{i}$ are included in the background model in their correct functional form. Omitting key variables can lead to biased estimates and compromise the accuracy of PVs (Bondarenko & Raghunathan, 2016; Meng, 1994). Therefore, it is recommended to include all variables in the background model that are used in subsequent analyses to provide unbiased population estimates (von Davier et al., 2009; Wu, 2005). When the background model is correctly specified, Sengewald and Mayer (2024) showed that estimates obtained by PVs are comparable to those from latent variable models.

## Accounting for Rapid Guessing in Plausible Value Estimation

While several approaches are available to account for RG in analyses of point estimates, the optimal strategy for handling RG in the context of PVs remains underexplored. Because relevant person-level variables can be incorporated into the background model, PVs offer a unique framework that can be leveraged to account for RG in ability estimation. The three threshold-based approaches of accounting for RG in PV estimation presented below aim to mitigate biases induced by RG by assuming that RG manifests at the response level. However, each of these models addresses RG at a distinct analytical level: either at the person level, at the response level, or both.

### Rapid Guessing as a Person-Level Characteristic

The *person-level model* conceptualizes RG as an individual characteristic measurable at the examinee level. This model builds upon the principle of PV estimation by incorporating a proxy for RG into the background model, facilitating adjustments for RG and its correlation with the estimated ability at the person level. RG at the person level can be operationalized using the response time effort (RTE; Wise & Kong, 2005), which describes an individual’s average engagement across the test. The idea is that each test consists of multiple person-item encounters. For each encounter, the test-taker decides whether to engage in SB or RG, and this decision is reflected in the response time. The overall RTE of individual *i* is calculated as

(6) $$\mathrm{RT}E_{i}=\frac{\sum_{j=1}^{J}\phi_{\mathrm{ij}}}{J},$$

with J describing the total number of items in the test and $\phi_{\mathrm{ij}}$ being the engagement indicator given in Equation 2. As the RTE is usually heavily skewed, it is common practice (e.g., Wise et al., 2021) to further dichotomize the RTE. Wise and Gao (2017) argue that, based on previous research, it can be assumed that ability estimates are considerably distorted when RG exceeds 10% of all provided responses. Therefore, test-takers can be defined as being engaged when they use SB on more than 90% of the items:

(7) $${\mathrm{RTE}}_{i}^{*}=\left\{ \begin{array}{c} 0,\;\mathrm{if}\;\mathrm{RT}E_{i}\leq .9\\ 1,\;\mathrm{if}\;\mathrm{RT}E_{i}\;>.9. \end{array}\right.$$

Buchholz and colleagues (2022) described the inclusion of a person-level RG operationalization as the simplest method for addressing this aberrant test-taking behavior in PV estimation. However, to our knowledge, no systematic analyses have been conducted to evaluate whether this approach can effectively attenuate biases induced by RG.

In summary, the person-level model accounts for RG in PV estimation by including the RTE as a proxy of RG in the background model. Thus, PVs are generated conditional on examinees’ overall test-taking effort, thereby adjusting population estimates for systematic differences in engagement.

### Rapid Guessing as a Response-Level Characteristic

The measurement model in the *response-levelmodel* corresponds to the effort-moderated model (EMM) by Wise and DeMars (2006). The EMM assumes that, for each person-item encounter, examinees decide whether to engage in SB or RG. Under SB, the probability of a correct response increases with ability and can be modeled using a standard IRT model. Under RG, by contrast, the probability of a correct response remains at the chance level, independent of ability. Thus, the functional relationship between ability and accuracy fundamentally differs depending on the response process. The EMM combines the two submodels representing SB and RG as

(8) $$P_{\mathrm{ij}}\left(\theta_{i}\right)=\left(\phi_{\mathrm{ij}}\right)\left(\mathrm{SB}\;\mathrm{model}\right)+\left(1-\phi_{\mathrm{ij}}\right)\left(\mathrm{RG}\;\mathrm{model}\right)$$

Depending on the engagement of examinee i for item j , which is determined by comparing the item response time to a threshold, either one of the two submodels is used to model the examinee’s response. Because the model assumes that responses given under RG are not psychometrically informative, rapid guesses are coded as missing in the IRT model, thereby reducing parameter bias compared with scoring them as incorrect (Rios & Deng, 2023). EMM scoring effectively handles RG but assumes RG occurs at random; if this assumption is violated, the accuracy of parameter estimates will be affected (DeMars, 2024; Rios & Soland, 2021a). Moreover, as RG rates increase, the standard errors of ability estimates also increase when using EMM scoring (Rios & Soland, 2021b). Despite these limitations, the EMM remains a practical and efficient method for handling RG (Liu et al., 2019; Rios & Deng, 2023).

In contrast to the person-level approach, the response-level model integrates RG directly into the measurement model. By coding rapid guesses as missing within the IRT framework, PVs are estimated conditional on effort at the response level, which allows for a fine-grained adjustment that accounts for response-level variation in engagement.

### Rapid Guessing as Person- and Response-Level Characteristic

The *combined model* addresses RG at both the person and response levels by combining the two previous approaches. It incorporates RG at the person level as a covariate into the background model for PV estimation while scoring the rapid guesses at the response level based on the EMM. Therefore, this approach utilizes the EMM as an established RG scoring method while also accounting for the correlation between RG and ability in PV estimation. Consequently, it combines the strengths of both the person-level and response-level models.

## Aims of the Study

The study aimed to investigate the impact of RG on group comparisons based on PVs and to evaluate different methods of accounting for RG in PV estimation. While PVs are widely used to adjust for measurement error and estimate population-level effects, the influence of RG on PVs has not been systematically explored. Therefore, this study seeks to (a) determine the bias introduced by RG in group comparisons based on PV estimates and (b) introduce and evaluate different approaches of handling RG in the estimation of PVs. To address these research aims, we present a simulation study that evaluates the performance of three approaches designed to account for RG in PV estimation on different analytical levels and compares them with an approach that does not account for RG. Furthermore, we demonstrate the application of these approaches using data from university students in a German LSA, providing insights into how these methods can improve the validity of group comparisons in LSAs.

## Simulation Study

### Design

The description of the simulation design follows the ADEMP (aim, data generation, estimands, methods, performance measures) structure suggested by Morris et al. (2019). The simulation was conducted in *R* (R Core Team, 2024). The analysis code, results, and supplemental material are provided at https://osf.io/gdnse/.

#### Aims

The simulation study aimed to evaluate the parameter recovery of group comparisons for different approaches of (not) accounting for RG in PV estimation.

#### Data Generation

**Figure 1.**
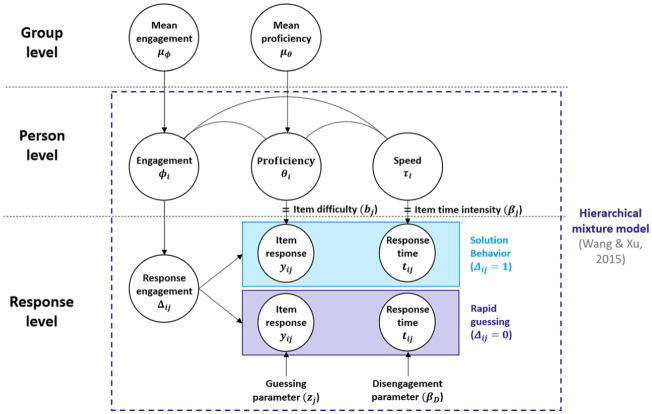
Hierarchical Mixture Model Used for Data Generation. *Note.* The figure depicts the hierarchical mixture model used for data generation in the simulation study. The model is adapted from C. Wang and Xu (2015; marked by dashed rectangle), who proposed a mixture modeling approach accounting for rapid guessing on the response level while still incorporating person-level characteristics and their relationships. It distinguishes between two latent response behavior classes: rapid guessing and solution behavior. Each class is characterized by different distributions of correct item responses and item response times. The current adaptation additionally incorporates a group level with aggregated engagement and proficiency values. The figure is adapted from Nagy and Ulitzsch (2022).

Data were generated based on the mixture hierarchical model by C. Wang and Xu (2015), as this model allows for individual differences not only in proficiency but also in test-taking engagement and speed while accounting for the relationship between these variables. The model assumes that test-takers decide for each item to engage either in SB or RG, represented by two latent classes on the response level. The probability of a correct response and the item response times each follow a mixed distribution, depending on the latent class. To accommodate the group comparison setting, the model was expanded in the current study at a group level (see Figure 1). The parameters were adapted from a previous simulation study on RG (Ulitzsch et al., 2020) to mirror empirical data of reading comprehension tests used in educational LSAs (e.g., Goldhammer et al., 2014; Welling et al., 2024).

Two groups were generated, with each group *g* consisting of $N_{g}=1,000$ participants. Within each group, the test-taking engagement $\phi_{i}$ , ability $\theta_{i}$ , and test-taking-speed $\tau_{i}$ of person *i* originated from a multivariate normal distribution with a mean vector $\mu =(\mu_{\phi_{g}},\mu_{\theta_{g}},\mu_{\tau })$ . Both $\mu_{\theta_{g}}$ and $\mu_{\phi_{g}}$ differed between the groups and were systematically varied between simulation conditions (see Table 1). In contrast, $\mu_{\tau }$ was zero for both groups across all conditions. The variance-covariance matrix for $\phi_{i}$ , $\theta_{i}$ , and $\tau_{i}$ was invariant across groups and defined as

(9) $$\Sigma =\left(\begin{array}{c} 1\\ .703.5\\ -.20-.200.05 \end{array}\right)$$

**Table 1.** Parameters Across Simulation Conditions

	Factor			Value			
Condition	Ability difference	RGR overall	RGR difference	$\mu_{\theta_{1}}$	$\mu_{\theta_{2}}$	RGR in group 1^a^	RGR in group 2^a^
1	0.50	5%	−5%	−0.25	0.25	7.5%	2.5%
2	0.50	5%	0%	−0.25	0.25	5.0%	5.0%
3	0.50	5%	5%	−0.25	0.25	2.5%	7.5%
4	0.50	15%	−5%	−0.25	0.25	17.5%	12.5%
5	0.50	15%	0%	−0.25	0.25	15.0%	15.0%
6	0.50	15%	5%	−0.25	0.25	12.5%	17.5%
7	1.00	5%	−5%	−0.50	0.50	7.5%	2.5%
8	1.00	5%	0%	−0.50	0.50	5.0%	5.0%
9	1.00	5%	5%	−0.50	0.50	2.5%	7.5%
10	1.00	15%	−5%	−0.50	0.50	17.5%	12.5%
11	1.00	15%	0%	−0.50	0.50	15.0%	15.0%
12	1.00	15%	5%	−0.50	0.50	12.5%	17.5%

*Note*. RGR = rapid guessing rate on response level, $\mu_{\theta_{1}}$ = mean ability in Group 1, $\mu_{\theta_{2}}$ = mean ability in Group 2.

a The RGRs were converted to the group mean engagement parameter $\mu_{\phi_{g}}$ as $1-\mathrm{RG}R_{g}=\frac{\exp(\mu_{\phi_{g}})}{1+\exp(\mu_{\phi_{g}})}$ .

The competence test was simulated to comprise 20 items. In the SB class *E*, the probability of a correct response followed a Rasch model (Rasch, 1960), thus depending on $\theta_{i}$ and the item difficulty $\xi_{j}$ of item *j*, with $\xi_{j}\in \{-1.0,-0.5,\;0.0,0.5,1.0\}$ . The log-transformed item response times followed a normal distribution with standard deviation $\sigma_{E}=0.30$ , mean $\beta_{j}-\tau_{i}$ , and item time intensities $\beta_{j}\in \{2.9,\;3.1,\;3.3,\;3.5,\;3.7\}$ corresponding to average item response times of 15 to 40 seconds. The five values of the item parameters $\xi_{j}$ and $\beta_{j}$ were each replicated four times to simulate the 20 items included in the test. In the RG class *D*, the probability of a correct response was defined for each item as $z_{j}=0.25$ , which resembles the guessing probability in multiple-choice questions with four response options. The log-transformed item response times followed a normal distribution with mean $\beta_{D}=1.25$ (≈3.5 seconds) and standard deviation $\sigma_{D}=0.60$ .

To evaluate the performance of the models in diverse conditions, three factors were systematically varied. First, the overall RG rate on the response level was varied between a lower (5%) and a higher (15%) rate. RG rates reported in the literature vary from under 1% to nearly 20%, depending on factors such as sample, test domain, and item or test characteristics (e.g., Michaelides & Ivanova, 2022; Ulitzsch et al., 2020). The RG rates in the present study were chosen to cover different scenarios while still being plausible for applied settings. Second, the size of the ability difference between the two groups was varied to correspond to a medium (0.5 *SD*) and a large (1.0 *SD*) gap. Similar values have been repeatedly found in educational studies (e.g., Bloom et al., 2008). Third, the difference in the RG rates between the two groups was varied, with (a) Group 1 having a lower RG rate than Group 2 ( $\Delta_{\%}=-5$ ), (b) both groups having the same RG rate ( $\Delta_{\%}=0$ ), and (c) Group 1 having a higher RG rate than Group 2 ( $\Delta_{\%}=5$ ). Since the impact of RG depends both on its frequency and the ability level of the (sub)sample, different RG rates in the two groups can bias the estimated aggregated abilities to varying degrees and thus also affect the estimation of ability differences. In total, the study comprised 2 (overall RG rate) × 2 (ability difference) × 3 (difference in RG rates) = 12 conditions. The specific parameters for each condition (i.e., $\mu_{\theta_{g}},\mu_{\phi_{g}}$ ) are displayed in Table 1.

#### Estimands

The main estimand is the ability difference between the two groups, measured by Cohen’s *d* (Goulet-Pelletier & Cousineau, 2018). To enhance the interpretation of the results, the mean ability $\mu_{\theta_{g}}$ and standard deviation of ability $\sigma_{\theta_{g}}$ of each group are included as supportive estimands.

#### Methods

For each simulated dataset, we estimated (a) the baseline model ignoring RG as well as (b) the person-level model, (c) the response-level model, and (d) the combined model that accounted for RG on the person level, response level, or both, respectively. As different approaches of accounting for RG vary in their sensitivity to distinct levels of misclassifications (Rios & Deng, 2023), models (b) to (d) were evaluated using two different methods to set the response-time thresholds: (a) the common-*k* method with a fixed threshold of 5 seconds for all items (*fixed threshold*) and (b) the visual-inspection method with an individual threshold for each item (*visual threshold*). Because it was not possible to visually determine a threshold for 20 items in all 12,000 simulated datasets, for each item parameter set (i.e., same item difficulty and time intensity) in each condition, one common threshold was determined by computing the minimum of the response time distribution between 5 and 15 seconds. The resulting 60 thresholds ranged between 5.54 and 12.86 seconds and were visually verified. The RTE was calculated and dichotomized using Equations 6 and 7, respectively.

For each dataset and method, the model with corresponding PVs was estimated in three steps using the package *TAM* (Robitzsch et al., 2024). First, a Rasch model (see Equation 1) was computed to obtain unbiased item difficulties. Then, the full model including the fixed item difficulties from step one and all covariates was estimated. As it is recommended to include all variables in the background model that are used in subsequent analyses (von Davier et al., 2009; Wu, 2005), the grouping variable was defined as a covariate for all models. In the person-level and combined models, the dichotomized RTE was included as an additional covariate to account for RG on the person level. Finally, 25 sets of PVs were drawn from the posterior distribution of the full model. Although the number of PVs provided in some educational LSAs is limited to 5 or 10, the use of more PVs can result in more efficient parameter estimates (see Laukaityte & Wiberg, 2017) and is common in LSAs such as the *National Assessment of Educational Progress*.

#### Performance Measures

As main performance measures, the rate of converged models, the absolute bias (as the relative bias is dependent on the true value and thus differs between conditions), and the empirical standard error were computed. In addition, the mean squared error (MSE) and the coverage are reported in the supplemental material. For each performance measure except the convergence criteria, the Monte Carlo standard error (MCSE) was computed (see Morris et al., 2019, p. 2086, for an overview of the performance measures with equations). The standard error of Cohen’s *d* was calculated using Equation 4 in Goulet-Pelletier and Cousineau (2018). All parameters were pooled over all PV sets using Rubin’s rules (Rubin, 1987). To obtain precise performance measures with small MCSEs, we chose a large number of simulated datasets, $N_{\mathrm{sim}}=1,000$ .

### Results

#### Threshold Diagnostics

There was a high agreement between the engagement estimated by the thresholds and the true engagement (>93% overlap for both thresholds and both RTEs in all conditions, see Tables S1 and S2 in the Supplemental Material). The visual threshold and the corresponding RTE exhibited a slightly better overlap than the fixed threshold and the corresponding RTE, especially in conditions with a high RG rate. The sensitivity (i.e., correctly classified engaged responses) was extremely high for both thresholds and RTEs (>98%), but the specificity (i.e., correctly classified rapid guesses) was higher for the visual threshold and corresponding RTE (>89%) than the fixed threshold and corresponding RTE (>72%) throughout all conditions.

#### Performance Measures

**Figure 2.**
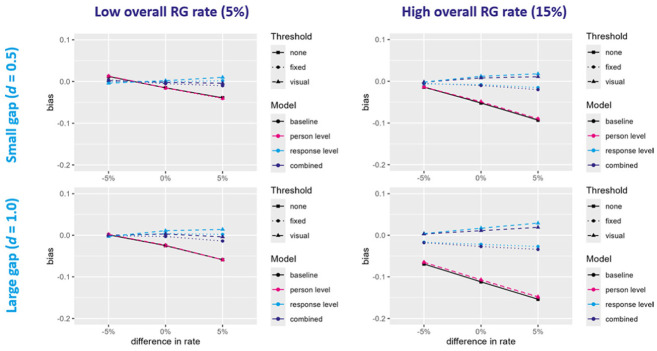
Absolute Bias in Estimated Ability Difference. *Note*. RG = rapid guessing, gap = true ability difference. The figure depicts the absolute bias in the estimated ability difference for (1) the baseline model that does not account for rapid guessing and (2) three proposed approaches of accounting for rapid guessing (person-level model, response-level model, combined model) using two threshold methods (fixed, visual). The 12 simulation conditions vary by rapid guessing rate, size of the true ability difference, and difference in rapid guessing rate.

All models converged in all conditions and simulated datasets. For all performance measures, estimands, models and conditions, the MCSE was estimated to be below 0.01 (see Tables 2 and 3 as well as Tables S3–S20 in the Supplemental Material). Overall, the bias in the estimated ability difference was rather low for most conditions and models (see Figure 2 and Table 2). The baseline model and the two person-level models tended to underestimate the ability difference, especially in the conditions with a high RG rate, a large true ability difference, and/or a higher RG rate in the high ability group than in the low ability group ( bias≥−0.16 ). The biases of the response-level and combined model were very small ( bias≤|0.04| ), independent of the threshold and in all conditions and models. The most pronounced bias occurred when the RG rate was high and the true ability difference large, with the models based on the fixed threshold slightly underestimating ( bias≥−0.04 ), and the models based on the visual threshold slightly overestimate the ability difference ( bias≤0.03 ). The empirical standard error of Cohen’s *d* ranged between 0.05 and 0.06 for all models and conditions (see Table 3).

**Table 2.** Absolute Bias of the Estimated Ability Difference With Monte Carlo Standard Errors

		Person-level model		Response-level model		Combined model	
Con.	Baseline model	Fixed	Visual	Fixed	Visual	Fixed	Visual
1	0.012 (0.002)	0.013 (0.002)	0.013 (0.002)	−0.001 (0.002)	−0.004 (0.002)	0.004 (0.002)	0.002 (0.002)
2	−0.015 (0.002)	−0.016 (0.002)	−0.015 (0.002)	−0.001 (0.002)	0.002 (0.002)	−0.005 (0.002)	−0.002 (0.002)
3	−0.039 (0.002)	−0.041 (0.002)	−0.040 (0.002)	0.002 (0.002)	0.010 (0.002)	−0.010 (0.002)	−0.004 (0.002)
4	−0.014 (0.002)	−0.013 (0.002)	−0.014 (0.002)	−0.006 (0.002)	−0.002 (0.002)	−0.005 (0.002)	−0.002 (0.002)
5	−0.052 (0.002)	−0.050 (0.002)	−0.049 (0.002)	−0.008 (0.002)	0.012 (0.002)	−0.010 (0.002)	0.008 (0.002)
6	−0.093 (0.002)	−0.090 (0.002)	−0.090 (0.002)	−0.015 (0.002)	0.018 (0.002)	−0.020 (0.002)	0.011 (0.002)
7	0.001 (0.002)	0.002 (0.002)	0.002 (0.002)	−0.002 (0.002)	−0.003 (0.002)	−0.001 (0.002)	0.000 (0.002)
8	−0.025 (0.002)	−0.025 (0.002)	−0.024 (0.002)	0.004 (0.002)	0.011 (0.002)	−0.003 (0.002)	0.003 (0.002)
9	−0.059 (0.002)	−0.059 (0.002)	−0.059 (0.002)	0.002 (0.002)	0.014 (0.002)	−0.014 (0.002)	−0.004 (0.002)
10	−0.069 (0.002)	−0.065 (0.002)	−0.065 (0.002)	−0.017 (0.002)	0.004 (0.002)	−0.018 (0.002)	0.003 (0.002)
11	−0.112 (0.002)	−0.108 (0.002)	−0.107 (0.002)	−0.022 (0.002)	0.017 (0.002)	−0.027 (0.002)	0.011 (0.002)
12	−0.154 (0.002)	−0.149 (0.002)	−0.148 (0.002)	−0.027 (0.002)	0.029 (0.002)	−0.034 (0.002)	0.019 (0.002)

*Note*. Con. = simulation condition. The terms “fixed” and “visual” refer to the threshold method used to flag rapid guesses. Displayed is the absolute bias and its Monte Carlo standard error in parentheses as described in Morris and colleagues (2019).

**Table 3.** Empirical Standard Error of the Estimated Ability Difference With Monte Carlo Standard Errors

		Person-level model		Response-level model		Combined model	
Con.	Baseline model	Fixed	Visual	Fixed	Visual	Fixed	Visual
1	0.053 (0.001)	0.052 (0.001)	0.052 (0.001)	0.052 (0.001)	0.052 (0.001)	0.052 (0.001)	0.052 (0.001)
2	0.055 (0.001)	0.055 (0.001)	0.055 (0.001)	0.055 (0.001)	0.055 (0.001)	0.055 (0.001)	0.055 (0.001)
3	0.055 (0.001)	0.055 (0.001)	0.055 (0.001)	0.056 (0.001)	0.056 (0.001)	0.055 (0.001)	0.056 (0.001)
4	0.053 (0.001)	0.052 (0.001)	0.053 (0.001)	0.053 (0.001)	0.054 (0.001)	0.053 (0.001)	0.054 (0.001)
5	0.052 (0.001)	0.053 (0.001)	0.053 (0.001)	0.053 (0.001)	0.053 (0.001)	0.054 (0.001)	0.053 (0.001)
6	0.052 (0.001)	0.052 (0.001)	0.052 (0.001)	0.053 (0.001)	0.054 (0.001)	0.053 (0.001)	0.054 (0.001)
7	0.058 (0.001)	0.058 (0.001)	0.058 (0.001)	0.057 (0.001)	0.057 (0.001)	0.057 (0.001)	0.057 (0.001)
8	0.054 (0.001)	0.054 (0.001)	0.054 (0.001)	0.054 (0.001)	0.054 (0.001)	0.054 (0.001)	0.054 (0.001)
9	0.053 (0.001)	0.053 (0.001)	0.053 (0.001)	0.054 (0.001)	0.054 (0.001)	0.054 (0.001)	0.054 (0.001)
10	0.052 (0.001)	0.052 (0.001)	0.052 (0.001)	0.053 (0.001)	0.054 (0.001)	0.053 (0.001)	0.053 (0.001)
11	0.053 (0.001)	0.054 (0.001)	0.054 (0.001)	0.056 (0.001)	0.057 (0.001)	0.056 (0.001)	0.056 (0.001)
12	0.052 (0.001)	0.052 (0.001)	0.053 (0.001)	0.055 (0.001)	0.057 (0.001)	0.055 (0.001)	0.057 (0.001)

*Note*. Con. = simulation condition. The terms “fixed” and “visual” refer to the threshold method used to flag rapid guesses. Displayed is the empirical standard error and its Monte Carlo standard error in parentheses as described in Morris and colleagues (2019).

The bias in the supportive estimands is displayed in Supplemental Figures S1 to S4 and Tables S3 to S6 in the supplemental material. The response-level models slightly overestimated the mean ability in the low ability group $\left(\mu_{\theta_{1}}\right)$ when the RG rate was high ( bias≤0.06) , while the baseline, person-level, and combined models barely showed any bias ( |bias|≤0.04 ). The baseline and person-level models underestimated the mean competence in the high ability group $\left(\mu_{\theta_{2}}\right)$ , especially when the RG rate was high, the true ability difference large, and/or when the high ability group exhibited a higher RG rate than the low ability group ( bias≥−0.20 ). The response-level and combined models performed better, with practically no bias in the condition with low RG rate. When the RG rate was high, the models based on the fixed threshold slightly underestimated $\mu_{\theta_{2}}$ , while the response-level model based on the visual threshold slightly overestimated $\mu_{\theta_{2}}$ ( |bias|≤0.06 ). All models slightly underestimated both standard deviations when the overall RG rate was high, most notably the baseline and person-level models and when the estimand was the standard deviation of the low ability group ( bias≥−0.12 ). The empirical standard error of $\mu_{\theta_{1}}$ and $\mu_{\theta_{2}}$ ranged between 0.03 and 0.04, and the empirical standard error of $\sigma_{\theta_{1}}$ and $\sigma_{\theta_{2}}$ between 0.02 and 0.03 (see Tables S7 to S10 in the Supplemental Material). The MSE and the coverage for all five estimands are reported in the Supplemental Material.

Overall, the models that accounted for RG on the response level (i.e., response-level and combined model) performed best, while only accounting for RG on the person level did not improve the performance compared to the baseline model. The differences between the models were most pronounced when the overall RG rate was high, the true ability difference was large, and/or the high ability group engaged in more RG than the low ability group. Especially in these conditions, the performance of the response-level and combined model also depended on the threshold method.

## Empirical Application

To illustrate the different models and investigate differences in conclusions that may be observed in practice, the same models as in the simulation study were used to compare groups of (former) university students in different subject areas of a German LSA.

### Methods

#### Sample

The sample consisted of 478 former university students who participated in the German *National Educational Panel Study* (NEPS; Blossfeld & Roßbach, 2019) that follows different cohorts across their life course. The participants (43% female) gave written informed consent and were currently or have been previously enrolled in different study subjects. For the group comparison, the sample was divided into two groups covering different study fields with distinct mean reading ability levels. Group 1 consisted of 302 participants (27% female) who were (formerly) enrolled in sports or engineering (age: *M* = 27.99, *SD* = 3.04), and Group 2 consisted of 176 participants (70% female) who were (formerly) enrolled in (veterinary) medicine or arts (age: *M* = 28.15, *SD* = 4.26).

#### Instrument

The participants took part in an unproctored, web-based test measuring reading comprehension. The test was specifically developed for the NEPS (Gehrer et al., 2013) and comprised 21 items with different response formats distributed over five reading tasks. However, to be comparable with the simulation study, only the 14 multiple-choice items were used in the present analysis. Each of these items contained four response options with one correct solution each (i.e., 25% chance level of a correct response).

#### Analysis

The response time data were preprocessed with R code developed by Welling et al. (2024). Item response time was defined as the time the participant spent in total on the respective item page (if the participant visited the page more than once, the different visit times were summed). As in the simulation study, a fixed and a visual threshold were set for each item. Because the computational approach for determining the visual threshold (see design section of the simulation study) could not identify clear thresholds for most items, the histograms of these item response times were visually scanned to find the intersection of the bimodal distribution. In case of ambiguous distributions, we followed Rios’ (2022) suggestion and preferred a liberal threshold. Consequently, the visual thresholds varied between 5 and 10 seconds (see Table 4).

**Table 4.** Descriptives by Item in the Empirical Example

			Valid responses	Response time	Fixed threshold		Visual threshold	
Item	Task	Position	*N*	*M* (*SD*)	Threshold	% RG	Threshold	% RG
1	1	1	477	29.29 (17.61)	5.00	0.2	5.00	0.2
2	1	2	477	35.53 (17.54)	5.00	0.8	7.00	1.1
3	1	3	477	26.93 (14.32)	5.00	1.5	6.00	2.1
4	2	6	466	27.00 (20.12)	5.00	1.7	8.00	2.4
5	2	7	466	22.29 (12.84)	5.00	1.9	7.00	2.2
6	3	10	463	27.71 (14.26)	5.00	2.4	9.00	3.7
7	3	11	463	21.22 (13.35)	5.00	3.0	6.00	3.2
8	3	12	460	31.06 (19.81)	5.00	3.3	6.00	3.3
9	3	13	460	34.62 (18.79)	5.00	3.5	10.00	3.9
10	3	15	450	27.14 (12.51)	5.00	3.6	8.00	4.0
11	4	16	424	32.13 (15.53)	5.00	3.8	9.02	5.4
12	4	18	396	32.93 (17.65)	5.00	5.6	9.12	6.6
13	5	19	358	22.97 (11.13)	5.00	4.5	6.79	5.3
14	5	20	348	18.36 (17.74)	5.00	4.9	5.00	4.9

*Note*. Response time and thresholds are specified in seconds.

Before model estimation, measurement invariance between the two groups was examined by testing for differential item functioning (DIF) for each dataset (i.e., the original data and for each threshold approach one dataset with missing values for all responses flagged as rapid guesses). To evaluate DIF, we examined whether item difficulties differed significantly between the two groups. In addition, we compared the DIF model to a model without DIF using information criteria.

The background models for the PV estimation were chosen to resemble the simulation study and included only the grouping variable in all models and the dichotomized RTE in the person-level and combined model. As in the simulation study, seven models were computed: a baseline model that ignores RG, and six models accounting for RG by combining three modeling approaches (person-level, response-level, combined) with two threshold-setting methods (fixed threshold, visual threshold). The computation of the models, the drawing and pooling of PV sets, and the estimation of all five outcomes ( $d,\mu_{\theta_{1}},\mu_{\theta_{2}},\sigma_{\theta_{1}},\;\sigma_{\theta 2}$ ) followed the same procedure as in the simulation study. Analyses were conducted in *R* (R Core Team, 2024). The raw data are available at NEPS Network (2024), while the analysis code, analysis results, and supplemental material are provided at https://osf.io/gdnse/.

### Results

There was no DIF between the two groups (see Tables S21 and S22 in the supplement). In total, 7.5% of the responses were missing, resulting in 6,185 valid item responses provided by the participants (Group 1: 3,895 responses, Group 2: 2,290 responses).

In the whole sample, 2.8% of the responses were identified as rapid guesses and 5.7% of the participants were classified as disengaged (i.e., ≥10% rapid guesses) when using the fixed threshold, compared to 3.3% rapid guesses and 6.7% disengaged participants when using the visual threshold. The RG rates differed between the groups: in Group 1, 3.5% (fixed) or 4.0% (visual) of the responses were identified as rapid guesses and 6.3% (fixed) or 7.6% (visual) of the examinees classified as disengaged, while in Group 2, only 1.6% (fixed) or 2.2% (visual) of the responses were rapid guesses and 4.6% (fixed) or 5.1% (visual) of the participants were disengaged. In total, 32.0% (fixed) or 34.1% (visual) of the responses flagged as rapid guesses were solved correctly, thus exceeding the chance level of 25% only slightly.

Overall, the estimated parameters and their confidence intervals did not vary considerably between the different models (see Table 5). The baseline model that ignored RG estimated the ability difference between the groups to be d=0.66 , 95%CI[0.42,0.90] . In all six models that accounted for RG, the ability difference was estimated to be slightly lower, ranging between d=0.59 , 95%CI[0.36,0.82 ], in the person-level model using the fixed threshold and d=0.64 , 95%CI[0.40,0.87] , in the combined model using the visual threshold. The estimated mean ability of Group 1 ( $\mu_{\theta_{1}}$ ) ranged between 0.10 in the person-level model and 0.16 in the response-level model, both models using the visual threshold. In contrast, the estimated mean ability of Group 2 ( $\mu_{\theta_{2}})$ ranged from 0.54 for the person-level model to 0.60 for the response-level model, using the fixed and visual threshold, respectively. The standard deviation in Group 1 ( $\sigma_{\theta_{1}}$ ) was estimated to be 0.70 in the baseline and person-level models, and to be 0.73 in both combined models. The estimated standard deviation in Group 2 ( $\sigma_{\theta_{2}})$ ranged between 0.73 for the baseline model and 0.76 for the combined model using the fixed threshold.

**Table 5.** Estimated Parameters in the Empirical Example

No	Model	Threshold	Cohen’s *d*	$\mu_{\theta_{1}}$	$\mu_{\theta_{2}}$	$\sigma_{\theta_{1}}$	$\sigma_{\theta_{2}}$
**1**	**Baseline**		0.66 [0.42, 0.90]	0.11 [0.01, 0.21]	0.58 [0.45, 0.71]	0.70 [0.63, 0.77]	0.73 [0.63, 0.84]
**2**	**Person-level**	fixed	0.59 [0.36, 0.82]	0.12 [0.02, 0.23]	0.54 [0.40, 0.69]	0.70 [0.62, 0.78]	0.74 [0.64, 0.83]
**3**		visual	0.63 [0.36, 0.91]	0.10 [–0.02, 0.23]	0.56 [0.42, 0.70]	0.70 [0.64, 0.77]	0.75 [0.65, 0.84]
**4**	**Response level**	fixed	0.60 [0.35, 0.84]	0.14 [0.02, 0.25]	0.57 [0.42, 0.72]	0.72 [0.64, 0.79]	0.75 [0.65, 0.85]
**5**		visual	0.60 [0.36, 0.84]	0.16 [0.05, 0.26]	0.60 [0.44, 0.75]	0.72 [0.65, 0.79]	0.76 [0.66, 0.85]
**6**	**Combined model**	fixed	0.63 [0.40, 0.86]	0.12 [0.01, 0.24]	0.59 [0.45, 0.73]	0.73 [0.65, 0.81]	0.77 [0.66, 0.87]
**7**		visual	0.64 [0.40, 0.87]	0.12 [0.02, 0.22]	0.59 [0.45, 0.73]	0.73 [0.65, 0.81]	0.75 [0.65, 0.85]

*Note. $\mu_{\theta_{1}}$* = estimated mean reading comprehension in Group 1, $\mu_{\theta_{2}}$ = estimated mean reading comprehension in Group 2.

$\sigma_{\theta_{1}}$ = estimated standard deviation in Group 1, $\sigma_{\theta_{2}}$ = estimated standard deviation in Group 2.

The results of the empirical example mostly reflect the finding from the simulation study for the condition with a low overall RG rate, a higher RG rate for the low ability group, and a moderate ability difference. As all confidence intervals overlap, there seems to be no pronounced bias in the five outcomes when (not) accounting for RG, resulting in similar estimates for all examined models.

## Discussion

Ability estimates can be seriously impaired if respondents do not invest motivation and effort in an administered competence test but engage in RG behavior (e.g., DeMars et al., 2013; Osborne & Blanchard, 2011; Wise & DeMars, 2010). Previous research, however, primarily focused on the consequences of RG on point estimates of ability, whereas its effect on PV has not yet been systematically explored. The present study filled this gap by determining the influence of RG on group comparisons based on PVs and by introducing and evaluating three different approaches of accounting for RG in PV estimation.

The results of the simulation study showed that the bias in the estimated ability difference varied across models, thresholds, and conditions, highlighting the importance of both model and threshold selection. When the RG rate was low, the corresponding bias in the estimated ability difference across all models was small. However, as the RG rates increased, the bias grew substantially. The models that ignored RG or accounted for RG only on the person level consistently underestimated the ability difference, particularly under a high RG rate, large true ability difference, and when the high ability group exhibited more RG than the low ability group. In contrast, models that accounted for RG on the response level, either alone or in combination with person-level adjustments, demonstrated substantially lower bias. Only when the RG rate was high, models based on a fixed threshold slightly underestimated the gap, while models using a visual threshold slightly overestimated it. In addition, the response-level models slightly overestimated the mean ability of the low ability group under a high RG rate, while the baseline, person-level, and combined models exhibited minimal bias. Conversely, the baseline and person-level models substantially underestimated the mean ability of the high ability group, particularly under a high RG rate and a large true ability difference. All models underestimated at least slightly the standard deviations when the RG rate was high, with the baseline and person-level model exhibiting the most pronounced bias. Across all outcomes and conditions, the combined model using the visual threshold (i.e., a low misclassification rate) performed best, although differences between the combined and response-level model were mostly marginal.

These findings indicate that ignoring RG in PV estimation can distort group comparisons of ability and may compromise the validity of inferences on educational inequalities. However, when the RG rate was small, the bias tended to be rather small. This aligns with previous research on point estimates of abilities, indicating that bias in group comparisons tends to be limited in many settings (Soland, 2018). A potential explanation for the small bias are group-level aggregation effects. As described by Wise et al. (2020), two mechanisms can reduce the impact of RG on aggregated scores. On the one hand, a diluting effect may occur if most respondents engage in SB, which reduces the impact of RG, especially when the RG rates are low. On the other hand, a cancellation effect can occur if individual score distortions vary in direction. As shown by these authors, some rapid guesses lead to negatively biased scores, while others result in positively biased scores (e.g., when the probability of a correct response is larger under RG than under SB), thereby offsetting each other in aggregated scores.

Furthermore, the findings of the simulation study suggest that the extent of the bias varies depending on the prevalence of RG in both groups and the size of the true ability difference. The results support key theoretical assumptions about bias caused by RG when not or inadequately accounted for. Bias increases (a) with higher RG rates, because a larger proportion of distorted responses is incorporated into the estimation process of PVs (Rios & Soland, 2021a), (b) in groups with higher competence since the probability of a correct response under SB and thus the difference between the probability of a correct response under SB vs. RG increases with proficiency (Rios et al., 2017), and (c) in group comparisons additionally when the more able group engages in more RG than the less able group, as a direct consequence of (a) and (b) (Anaya & Zamarro, 2023; Soland, 2018). The finding that the bias increased with the size of the true ability difference is attributable to the effect of (b): the larger the true difference, the greater was the simulated ability of the high ability group and thus the distorting effect of RG.

The evaluation of the different approaches of accounting for RG in PV estimation showed that adjusting for RG solely on the person level by including a proxy for RG yielded little improvement over the model that ignored RG, whereas the models accounting for RG on the response level effectively minimized bias. These findings suggest that also in PV estimation, RG should be considered at the response level, and are thus consistent with previous research on point estimates (Wise & DeMars, 2006). Leveraging the unique framework that offers PVs by including a proxy for RG as a covariate in the background model, as suggested by Buchholz and colleagues (2022), does not seem to suffice in minimizing the biases induced by RG. The person-level model operates under the assumption that response patterns accurately represent ability, an assumption that is violated under RG. As the individual posterior predictive distribution of PVs is based on both the measurement and the background model, adjustments in the background model may be insufficient if the likelihood in the measurement model is already distorted by responses that do not reflect true ability. Nevertheless, even the response-level models remained imperfect, particularly in estimating the ability of lower-performing groups, which tended to be overestimated. In these cases, combining person- and response-level adjustments provided the most accurate estimates by also addressing the relationship between RG and competence.

However, the performance of the models that were effective in accounting for RG (i.e., the response-level and combined models) varied more between the different threshold methods than between the different approaches used for accounting for RG. Notably, the combined model only yielded the best results when using a threshold method with a low misclassification rate. These findings suggest that the choice of threshold can also impact model performance in PV estimation and align with those of Rios and Deng (2023) on point estimates of ability. They highlight the importance of thorough threshold selection before choosing the correct model for accounting for RG.

The empirical study demonstrated that the proposed approaches of accounting for RG in PV estimation are both applicable and feasible in practice. In the sample used for the empirical application, all models of (not) accounting for RG yielded similar results. This is consistent with the findings from the simulation study for the condition with a low overall RG rate, a higher RG rate for the low ability group, and a moderate group difference—characteristics that were reflected in the actual sample. As the overall RG rate was even lower than under the low RG rate condition of the simulation study, these findings further support the notion that group comparisons do not seem to be heavily biased when RG rates are low.

### Implications for Group Comparisons in Large-Scale Assessments

The study demonstrates that accounting for RG in the estimation of PVs in LSAs can reduce the bias induced by RG and is feasible, as the evaluated models are computationally efficient and easy to implement. However, our findings indicate that the bias introduced by RG is influenced by the ability distribution across groups. Failure to account for RG poses a risk of misinterpreting ability differences, potentially leading to biased conclusions about educational outcomes. This, in turn, could have far-reaching implications for educational policies and decision-making, such as changes in the ranking of countries or demographic subgroups. Therefore, it is crucial to be aware of the potential bias introduced by RG and consider appropriate corrective measures when interpreting assessment outcomes.

### Limitations and Future Directions

The findings presented offer several opportunities for follow-up research. First, future research should examine whether including additional covariates related to test-taking engagement into the background model as proposed by Buchholz et al. (2022) can improve the adjustment for RG in PV estimation on the person level. For reasons of clarity, RTE as a proxy for test-taking engagement and the grouping variable were included as the only covariates in the current study. Although our simulation study showed that the RTE can reliably detect RG behavior (see Table 2 in the supplements) and thus explains a large part of the variance of test-taking engagement, it was not sufficient to adjust for the impact of RG on PV. Further studies could investigate whether an integration of a broader set of covariates, which explain an even greater part of the variance of test-taking engagement and ability, can improve the estimation of PVs.

Second, threshold selection plays a critical role in model performance, as different threshold methods can lead to significant variations in results (Rios & Deng, 2023). This study employed two well-established and easily applicable methods: the common-*k* method being practical and straightforward, and the visual-inspection method optimized for better performance. However, these methods also have their limitations in practice. The common-*k* approach disregards item-level variability, while the visual-inspection method can only detect RG when a bimodal distribution is present—an assumption that may not hold for items requiring relatively short response times under SB. Therefore, future studies could evaluate the performance of the approaches presented in this study also using different threshold techniques, for example, the normative-threshold method (by classifying all responses with a response time shorter than fixed percentage of the average response time as RG; Wise & Ma, 2012) or by incorporating accuracy information in threshold selection (e.g., Lee & Jia, 2014).

Third, to keep the scope and interpretation of the results in the simulation study comprehensible, the correlation between RG and ability was not varied in the present study and mean item response times were chosen to resemble the time demands of simple multiple-choice items. Based on previous research using empirical data (e.g., Silm et al., 2020), we selected a relatively strong correlation between RG and ability, recognizing its potential impact on model performance and bias in estimating ability differences. However, future studies should also take variations in the relationship between ability and RG into account and evaluate the performance of the models under different conditions. Moreover, they could examine whether different time demands of items influence the classification accuracy of the different threshold methods, thereby potentially impacting the performance of the models.

Fourth, the present study focused on easily applicable approaches of accounting for RG on different analytical levels, leveraging the unique framework provided by PVs and investigating whether adjusting for RG in the background model improves performance. Building on these findings, future research should consider exploring alternative modeling approaches, such as multidimensional IRT models (Liu et al., 2019; B. Wang et al., 2025) or specialized mixture models (Nagy & Ulitzsch, 2022; C. Wang & Xu, 2015). These models account for RG on the response level while factoring in the relationship of RG and ability and may thus offer improved accuracy in handling RG effects. Finally, incorporating further process data, such as item nonresponse and text-reread, could provide a more comprehensive understanding of test-taking behavior and enhance the precision of RG adjustments in LSAs (see Welling et al., 2024, for an example).

## Conclusion

This study examined the impact of RG on group comparisons based on PVs and evaluated different approaches of accounting for RG in PV estimation. While a simulation study systematically varied RG rates, true ability difference, and variations in RG prevalence between groups to assess whether the bias of RG on the group comparisons can be minimized by using different approaches of accounting for RG in PV estimation, an empirical study subsequently demonstrated that these approaches are applicable in practice. The results indicate that ignoring RG leads to systematic underestimation of ability differences, particularly when RG rates are high, the true ability difference is large, and the more able group engages in more RG. Models that accounted for RG at the response level, either alone or in combination with person-level adjustments, significantly improved estimation accuracy, though slight over- and underestimations of ability differences remained in some conditions. Person-level adjustments alone showed little advantage over the baseline model that ignored RG. Furthermore, the choice of threshold method influenced model performance, stressing the importance of thorough threshold selection.

These findings emphasize the necessity of incorporating RG adjustments in PV estimation to avoid biased group comparisons with potential consequences for educational policies and decision-making. They further indicate that accounting for RG solely on the person level with a proxy for RG does not suffice in attenuating the bias induced by RG. Future research should explore additional background covariates, refined thresholding techniques, and alternative modeling approaches, such as multidimensional IRT or integration of additional process data, to further improve RG adjustments in LSAs.
